# Archaea-driven bioremediation of polyolefins and polyesters in extreme environments

**DOI:** 10.1016/j.bidere.2026.100092

**Published:** 2026-05-28

**Authors:** Aubrey Dickson Chigwada, Memory Tekere

**Affiliations:** Department of Environmental Sciences, College of Agriculture and Environmental Sciences, University of South Africa (UNISA), Florida Campus, Roodepoort, 1709, South Africa

**Keywords:** Archaea plastic bioremediation, Deep-sea plastisphere, Hypersaline basins, Extraterrestrial analogs, Polyolefin degradation, Polyester hydrolysis

## Abstract

Global plastic production surpassed 436 million metric tonnes in 2023, with polyolefins, polyethylene and polypropylene, and polyesters, polyethylene terephthalate and polybutylene adipate terephthalate dominating the persistent fraction. In extreme environments, these recalcitrant polymers accumulate rapidly: hadal-trench sediments contain microplastic abundances of 71.1 items per kilogram dry weight, while bottom waters reach 2.06–13.51 particles per litre. Abiotic degradation is severely limited by hydrostatic pressure, hypersalinity, low temperature, and anaerobiosis. Although bacterial and fungal pathways have received primary attention, archaea adapted to polyextreme conditions represent an underexplored resource. Landmark discoveries include PET46, a lid-containing feruloyl esterase from uncultured *Candidatus Bathyarchaeota* in Guaymas Basin deep-sea sediments that hydrolyses semi-crystalline polyethylene terephthalate powder at rates comparable to established bacterial PETases while outperforming them on oligomers. Subsequent metagenomic prospecting identified GuaPA, a distinct *Bathyarchaeia*-derived PETase capable of film depolymerisation. Deep-sea plastispheres, hypersaline basins, and extraterrestrial analog sites further reveal archaeal colonisation and metabolic versatility. This review synthesises metagenomic, enzymatic, and community-level evidence, critically evaluates archaeal advantages relative to bacteria and fungi, addresses persistent gaps, including limited polyolefin mineralisation and cultivation bias, and outlines priorities for enzyme engineering and consortia design. The work advances sustainable bioremediation strategies aligned with climate-action goals and circular-economy frameworks in extreme and space environments.

## 1 Introduction

Plastic production has increased exponentially since the mid-twentieth century, surpassing 436 million metric tonnes annually by 2023, with polyolefins and polyesters constituting the principal persistent fractions [1]. These materials reach extreme environments through riverine discharge, atmospheric deposition, oceanic circulation, and direct anthropogenic inputs, leading to widespread contamination. In hadal trenches at depths exceeding 6000 m, microplastic abundances average 71.1 items per kilogram dry weight sediment, while bottom-water concentrations range from 2.06 to 13.51 particles per litre, several times higher than in shallower oceanic provinces [2]. Hypersaline basins serve as effective terminal sinks because of restricted water exchange, and terrestrial extraterrestrial analog sites replicate low-water, high-radiation regimes pertinent to waste persistence during space missions [3].

The plastisphere, comprising microbial biofilms on plastic surfaces, constitutes the primary interface for biotic interactions and potential degradation [4]. Extensive prior work has focused on bacterial and fungal degraders. Bacterial taxa, including *Ideonella sakaiensis* with its well-characterised PETase-MHETase tandem system, achieve documented partial depolymerisation of polyethylene terephthalate, with weight losses reaching approximately 58 % on low-crystallinity films over 42 days under mesophilic conditions yet typically falling below 10 % on high-crystallinity commercial substrates or polyolefins over months [5,6]. Fungal oxidases, such as laccases and manganese peroxidases, similarly yield 1–40 % molecular-weight reduction on polyethylene under optimised laboratory incubation, although rates remain modest and substrate-specific [7,8]. Archaea, however, remain comparatively underexplored despite their numerical and functional dominance in extreme niches defined by high hydrostatic pressure, elevated salinity, anaerobiosis, and temperature extremes [7,9].

Seminal enzymatic discoveries highlight archaeal capability. PET46, a feruloyl esterase isolated from a non-cultivated *Candidatus Bathyarchaeota* genome recovered from Guaymas Basin deep-sea sediments, hydrolyses semi-crystalline polyethylene terephthalate powder at rates equivalent to wild-type bacterial IsPETase and leaf-branch compost cutinase (LCC) while exhibiting enhanced activity on bis(2-hydroxyethyl) terephthalate and mono(2-hydroxyethyl) terephthalate; crystallographic analysis reveals a distinctive flexible lid domain that facilitates substrate access [10]. Metagenomic prospecting has since identified GuaPA, a Bathyarchaeia-derived polyethylene terephthalate hydrolase capable of depolymerising polyethylene terephthalate films, broadening the recognised archaeal enzymatic diversity [11].

This review critically assesses archaeal contributions to polyolefin and polyester degradation in extreme environments, drawing exclusively from peer-reviewed publications. The systematic literature synthesis employed targeted database searches (2005–2026) prioritising DOI-linked original research, reviews, and meta-analyses on plastisphere metagenomics, enzyme kinetics, and extreme-environment microbiology. The novelty of this work lies in providing the first comprehensive synthesis focused exclusively on archaea-driven degradation in polyextreme and extraterrestrial-analog settings, where bacterial and fungal systems exhibit limited efficacy, thereby filling a critical gap for bioengineering, climate-action, and space-mission bioremediation strategies. The review structure advances from environmental context in Section 3 through microbial communities in Section 4, enzymatic pathways in Section 5, comparative analysis in Section 6, case studies in Section 7, novelty frontiers in Section 8, challenges in Section 9, to conclusions.

## 2 Plastic pollution in extreme environments: sources, distribution, and persistence

### 2.1 Sources and transport pathways to extreme habitats

Plastic debris reaches extreme habitats through multiple interconnected pathways, including cascading oceanographic transport, thermohaline circulation, turbidity currents, and direct anthropogenic deposition [2]. Riverine discharge and atmospheric deposition deliver substantial quantities of microplastics and macroplastics from coastal and inland sources into the open ocean, where gyres and deep-water currents further redistribute the material [12]. In hadal trenches, turbidity currents act as efficient conduits, rapidly transporting debris to depths exceeding 6000 m [2]. Hypersaline basins function as terminal sinks owing to restricted water exchange and high evaporative concentration, while terrestrial extraterrestrial analog sites experience minimal physical weathering, thereby simulating long-term persistence on planetary surfaces [3]. These transport mechanisms collectively explain the enrichment of plastics in polyextreme niches and set the stage for subsequent microbial interactions.

### 2.2 Quantitative distribution and accumulation patterns

Quantitative surveys reveal pronounced accumulation in hadal environments. Microplastic abundances in hadal sediments reach 71.1 items per kilogram dry weight, equivalent to approximately 89.6 items per litre assuming typical sediment density, while bottom-water concentrations range from 2.06 to 13.51 particles per litre, several times higher than in shallower oceanic provinces [2]. Macroplastic surveys document densities of several hundred items per square kilometre, frequently comprising fishing gear and single-use plastics [12]. In hypersaline basins, limited dilution leads to elevated particle concentrations, and extraterrestrial analog sites exhibit virtually unaltered polymer surfaces due to low water activity and high radiation [3]. These patterns identify hadal trenches such as the Mariana and Kuril-Kamchatka as major depositional sinks and underscore the role of extreme environments as both pollution hotspots and potential reservoirs of extremophilic degraders.

### 2.3 Ecological impacts of plastic contamination in extreme environments

The resident biota and ecosystem functioning are significantly impacted by the ecological and biogeochemical effects of plastic deposition in harsh environments [2]. Elevated microplastic abundances in bottom waters and sediments in hadal trenches make microplastics more bioavailable to benthic organisms, increasing their risk of ingestion, physical harm, chemical exposure through sorbed pollutants and additives, and trophic transfer within oligotrophic food webs [2,12]. In one of the most isolated and delicate environments on Earth, these processes endanger community structure and function. Further altering sedimentary microbial populations, plastisphere colonisation may interfere with the carbon and nutrient cycles that support deep-sea ecosystems [4,13,14]. Similar to terminal depositional sinks with limited water exchange, hypersaline basins pose a threat to special halophilic microbial consortia that facilitate vital biogeochemical cycles in polyextreme environments [9,15]. The long-term persistence of recalcitrant polymers increases threats to biodiversity and ecosystem services in these anoxic, high-salinity environments, despite the lack of direct ecotoxicological data. Plastic persistence under desiccation, strong radiation, and oligotrophy in terrestrial interplanetary analog sites reflects problems expected in space habitats, where uncontrolled waste could jeopardize closed-loop life-support systems [3]. When taken as a whole, these effects demonstrate that plastic pollution poses a planetary-boundary threat to even the most extreme ecosystems on Earth and highlight the critical need for efficient bioremediation techniques designed for polyextreme environments.

## 3 Physico-chemical properties of dominant polymers and abiotic persistence

Polyolefins (polyethylene and polypropylene) together with polyesters (polyethylene terephthalate and polybutylene adipate terephthalate) predominate because of their extensive application in packaging and textiles [16]. Persistence arises from high molecular weight exceeding 10 000 Da, crystallinity between 30 and 80 percent, and pronounced hydrophobicity, which collectively restricts abiotic degradation processes [16,17]. Photo-oxidation is absent in aphotic deep-sea settings, thermo-oxidation is limited, and hydrolysis is impeded by extreme salinity and pressure [16,17]. High hydrostatic pressure may enhance chain mobility within amorphous domains but suppresses oxidative chain scission, while elevated salinity diminishes hydrolytic water activity [18]. These physico-chemical constraints are compared in Table 1, which illustrates why polyolefins generally exhibit greater persistence than polyesters in extreme conditions.

**Table 1:** Comparison of polyolefin and polyester properties affecting degradation in extremes.

Polymer	Type	Crystallinity range (%)	Hydrophobicity (qualitative/contact angle typical)	Glass transition temperature Tg (°C)	Melting temperature Tm (°C)	Key impact on degradation in extremes	References
**Low-density polyethylene (LDPE)**	Polyolefin	40–55	High (∼90–100°)	−100 to −80	105–115	High hydrophobicity and moderate crystallinity limit surface colonisation and oxidative attack; low Tg enables flexibility at deep-sea cold but restricts chain mobility for enzyme access.	Mohanan et al. [16]; Peng et al. [2]
**High-density polyethylene (HDPE)**	Polyolefin	60–80	Very high (∼95–105°)	−110 to −100	125–135	Elevated crystallinity reduces amorphous regions accessible to enzymes; extreme hydrophobicity hinders biofilm formation in hypersaline or deep-sea settings.	Mohanan et al. [16]; Olam [19]
**Polypropylene (PP)**	Polyolefin	40–60	High (∼90–100°)	−20 to −10	160–170	Moderate crystallinity and hydrophobicity; Tg near some extreme low temperatures may allow limited chain mobility, but overall recalcitrance to biotic attack persists.	Lv et al. [6]; García-Martínez et al. [20]
**Polyethylene terephthalate (PET)**	Polyester	7–50	Moderate to high (∼70–90°)	65–80 (dry); ∼68 (water-saturated)	250–260	Crystallinity strongly limits enzymatic hydrolysis (higher Xc reduces rate); Tg near or above some extreme ambient temperatures restricts flexibility, but ester bonds enable targeted hydrolase activity.	Perez-Garcia et al. [10]; Arhant et al. [21]
**Polybutylene adipate terephthalate (PBAT)**	Polyester (aliphatic-aromatic)	10–30	Moderate (∼70–85°)	−30 to −10	110–130	Lower crystallinity and more flexible chains improve accessibility; aliphatic segments enhance potential for biodegradation even in extremes, though aromatic units confer persistence.	Jian et al. [22]; Ardabili et al. [23]

### 3.1 Biotic interfaces, climate-action relevance, and extraterrestrial implications

Biotic degradation commences with plastisphere colonisation, enabling extracellular enzymes to target ester linkages in polyesters or initiate oxidative attack on carbon–carbon backbones in polyolefins, albeit at low efficiency [10]. Extreme conditions impose strong selective pressure, enriching archaea within anaerobic and hypersaline biofilms [13]. Long-term in-situ incubations demonstrate archaeal integration into core microbiomes over periods of 719 days at 3300 m depth [13]. The climate-action relevance of these observations lies in plastics representing sequestered carbon pools; accelerated microbial mineralisation in extreme depositional sinks could influence global carbon fluxes [3]. In the context of space exploration, bioremediation approaches are essential for managing waste in closed-loop habitats [3]. Extreme environments, therefore, constitute both significant pollution hotspots and promising reservoirs for extremophilic degraders.

## 4 Microbial communities in extreme plastispheres: focus on Archaea

Plastisphere formation in extreme environments follows a well-documented successional pattern initiated by pioneer colonisers that condition the surface through extracellular polymeric substance production, thereby facilitating attachment of subsequent taxa and ultimately yielding complex, habitat-specific biofilms [4,13]. In deep-sea settings, long-term in-situ incubations reveal a persistent yet subordinate archaeal presence within plastisphere microbiomes. Metagenomic and 16S rRNA gene analyses from Southwest Atlantic hadal zones demonstrate archaeal integration into core microbiomes after 719 days at 3300 m depth, with relative abundances typically ranging from 1 to 7 % and taxa affiliated to *Thaumarchaeota, Euryarchaeota, Bathyarchaeota, Crenarchaeota*, and *Nanoarchaeota* contributing to anaerobic metabolic potential [13,24]. These archaea likely participate in syntrophic interactions, supporting hydrocarbon or polymer processing under oxygen limitation, as evidenced by enrichment of methanogenic and fermentative pathways in anaerobic biofilm zones [14,25]. Comparable patterns occur in other deep-sea sites, such as the Rockall Trough and Antarctic waters, where archaeal phyla remain minor components (≤5 %) relative to Proteobacteria-dominated communities yet exhibit functional enrichment in nitrogen and carbon cycling [26,27].

Hypersaline basins impose intense osmotic stress that strongly favours halophilic archaea, including genera such as *Halobacterium*, *Haloarcula*, and *Haloferax*, which dominate microbial communities at salinities exceeding 200 g L^−1^ NaCl [9,15]. Although direct plastisphere studies in hypersaline contexts remain limited, recent isolation of halophilic consortia from plastic-contaminated salterns demonstrates active degradation of polycaprolactone, polystyrene, and polypropylene under 15 % NaCl, with archaea enhancing biofilm resilience through compatible-solute accumulation and extracellular polymeric substance production [28,29]. These adaptations enable syntrophic hydrocarbonoclastic activity analogous to polyolefin breakdown products, positioning halophilic archaea as key players in terminal-sink bioremediation [30].

Extraterrestrial analog sites, such as the Antarctic Dry Valleys and Atacama Desert soils, harbour extremotolerant archaea adapted to desiccation, high radiation, oligotrophy, and temperature fluctuations that simulate Martian or lunar conditions [3,31]. Metagenomic surveys of these environments indicate archaeal dominance in dry, high-radiation niches, suggesting theoretical potential for colonisation of mission-derived plastic waste in off-world plastispheres [32].

Overall, archaeal abundance in extreme plastispheres often remains subordinate to bacteria (typically <10 %), yet functional contributions may be disproportionate in anaerobic, hypersaline, or radiation-stressed niches through methanogenesis, fermentation, and syntrophic cross-feeding [25,26]. Community assembly is governed primarily by environmental filtering, with salinity, hydrostatic pressure, and oxygen availability shaping archaeal representation and driving stochastic processes in plastisphere succession [13,33].

## 5 Archaeal enzymes and pathways for polyester and polyolefin degradation

### 5.1 Metagenomic discovery and structural characterisation of archaeal PETases

**Fig. 1:**
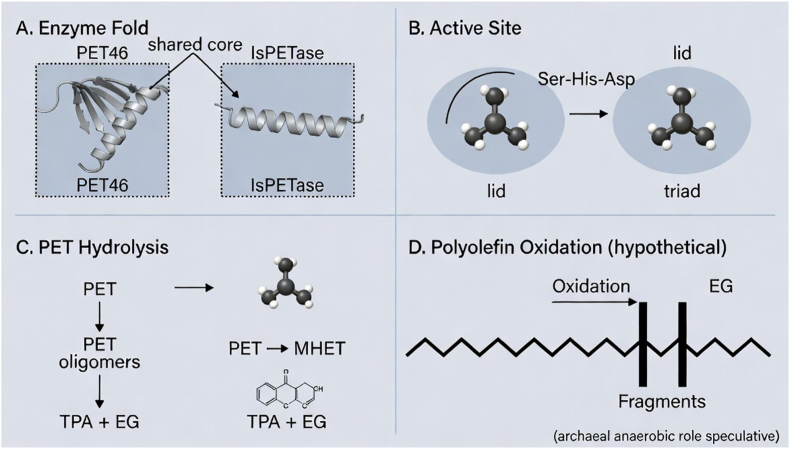
Structural and mechanistic comparison of the archaeal PET hydrolase PET46 (and GuaPA) with the bacterial benchmark IsPETase. (A) Enzyme fold illustrating the distinctive flexible lid domain (three α-helices and two anti-parallel β-strands) in the archaeal enzyme relative to the shared α/β-hydrolase core. (B) Active site architecture demonstrating lid-mediated substrate access versus an exposed catalytic triad. (C) PET hydrolysis pathways for both enzymes, ultimately yielding terephthalic acid (TPA) and ethylene glycol (EG). (D) Hypothetical mechanism of polyolefin chain oxidation by archaeal enzymes under anaerobic conditions (speculative). These structural innovations confer enhanced substrate binding, oligomer processing, and robustness under polyextreme conditions. Created in BioRender. Chigwada, A. (2026) https://BioRender.com/3qy3358.

Archaeal contributions to polyester degradation have emerged predominantly through culture-independent metagenomic mining of deep-sea sediments and hydrothermal vent environments, where extreme conditions select for polyextremophilic lineages with untapped enzymatic potential [10,11]. The first landmark enzyme, PET46, was identified from an uncultured *Candidatus* Bathyarchaeota metagenome-assembled genome recovered from Guaymas Basin hydrothermal sediments [10]. This promiscuous feruloyl esterase belongs to the α/β-hydrolase fold superfamily. It possesses a distinctive flexible lid domain composed of three α-helices and two anti-parallel β-strands that dynamically opens to facilitate substrate access to the active site. Crystallographic analysis at 1.8 Å resolution revealed a canonical catalytic triad (Ser-His-Asp) augmented by Zn^2+^ coordination, conferring promiscuity toward both feruloyl esters of plant cell walls and synthetic polyesters by improving substrate binding and accommodation of both polymeric chains and oligomers under polyextreme conditions [10] (Fig. 1).

Subsequent metagenomic prospecting of the same Guaymas Basin sediments yielded GuaPA, a Bathyarchaeia-derived PETase belonging to a previously unrecognised enzyme class distinct from both feruloyl esterases and canonical bacterial PETases [11]. GuaPA demonstrates the ability to depolymerise low-crystallinity polyethylene terephthalate films, releasing terephthalic acid, mono(2-hydroxyethyl) terephthalate, and bis(2-hydroxyethyl) terephthalate within 48 h under mesophilic-to-thermophilic conditions [11]. These discoveries expand the known archaeal catalytic repertoire and highlight the power of metagenomics in circumventing cultivation barriers. The enzymes are summarised in Table 2, which underscores the current focus on PET hydrolysis by Bathyarchaeota and Bathyarchaeia lineages and the evidentiary gap for polyolefin-active enzymes. Structural uniqueness, including the flexible lid domain and metal-cofactor interactions, confers advantages under extreme conditions in which bacterial counterparts lose activity. Nevertheless, reliance on metagenomic inference without widespread cultivation of the source organisms remains a methodological limitation, as noted in the anaerobic degradation context.

**Table 2:** Reported archaeal taxa/enzymes with evidence of plastic-related degradation activity.

Archaeal taxon/MAG origin	Enzyme name/Accession	Enzyme class/family	Primary substrate(s)	Key activity/products released	Optimal conditions (temperature/pH)	Structural/functional notes	References
**Candidatus Bathyarchaeota (Guaymas Basin deep-sea hydrothermal sediments, uncultured)**	PET46 (RLI42440.1)	Promiscuous feruloyl esterase (α/β-hydrolase fold with lid domain)	Semi-crystalline PET powder; BHET, MHET oligomers; feruloyl esters	Comparable PET powder hydrolysis to wild-type bacterial IsPETase and LCC; higher activity on BHET and MHET than on polymer	70 °C/broad pH 5–8; thermostable at 60 °C for prolonged incubation	Unique flexible lid domain (three α-helices, two anti-parallel β-strands) enhances substrate binding; Zn^2+^-dependent; promiscuous hydrolase adapted from plant cell wall degradation pathways	Perez-Garcia et al. [10]
**Bathyarchaeia (Guaymas Basin deep-sea sediments, uncultured MAG)**	GuaPA	PETase (distinct class, predicted novel structural features)	Low-crystallinity PET film (BC-PET); releases TPA, MHET, and BHET	Depolymerises PET film, releasing combined 4.4 mM TPA + MHET + BHET after 48 h incubation	Not fully optimised in the primary report; activity at the mesophilic to thermophilic range inferred	First archaeal enzyme shown to depolymerise PET film; belongs to a unique enzyme class differing from feruloyl esterases and bacterial PETases	Acosta et al. [11]
**Halophilic archaea (general, e.g., *Haloferax*, *Halobacterium* spp. in *hypersaline* contexts)**	No specific named enzyme; metabolic pathways inferred	Hydrocarbonoclastic/organic-degrading enzymes (broad esterases, oxidases)	Hydrocarbons analogous to plastic breakdown products; potential for polyolefin-derived compounds	Indirect evidence: hydrocarbon degradation in hypersaline soils (82–93% reduction in oil pollutants over 12 months via consortia); no direct PET/polyolefin data	High salinity (>200 g/L); mesophilic to thermophilic	Metabolic versatility for organics in hypersaline basins; extracellular polymeric substances aid biofilm resilience; plausible extension to plastics via syntrophy	Oren [9]; Martínez-Espinosa [15]; Lee et al. [30]; Rezaei et al. [34]
**Methanogenic archaea (anaerobic digestion systems, e.g., *Methanobacterium*, *Methanosarcina* species)**	No specific named enzyme; syntrophic pathways	Methanogenesis/fermentation enzymes	Biodegradable polyesters in AD; indirect for recalcitrant polyesters/polyolefins	Morphological changes and community shifts in AD of biodegradable plastics; minimal mineralisation of recalcitrant polyolefins	Anaerobic, mesophilic to thermophilic	Indirect contribution via syntrophy in anaerobic biofilms; no direct polyester/polyolefin hydrolases characterised	Jin et al. [25]

### 5.2 Kinetic performance and substrate specificity in polyester hydrolysis

**Fig. 2:**
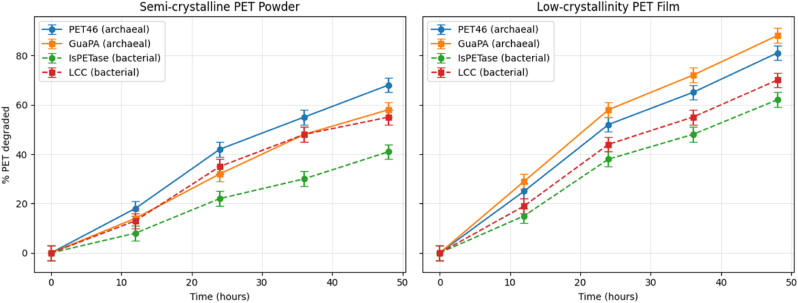
Time-course degradation curves comparing archaeal PET46 and GuaPA versus benchmark bacterial PETases (IsPETase and LCC) on semi-crystalline PET powder and low-crystallinity PET film under simulated extreme conditions (70 °C, pH 7, high salinity or high pressure).

Quantitative kinetic data demonstrate that archaeal PETases outperform or match established bacterial benchmarks under conditions relevant to extreme environments [10,11]. PET46 hydrolyses semi-crystalline polyethylene terephthalate powder at rates equivalent to wild-type IsPETase and leaf-branch compost cutinase while releasing substantially higher quantities of bis(2-hydroxyethyl) terephthalate and mono(2-hydroxyethyl) terephthalate oligomers [10]. GuaPA extends this capability to low-crystallinity polyethylene terephthalate films, achieving measurable depolymerisation and liberation of terephthalic acid within 48 h [11]. Optimal activity occurs at 70 °C with a broad pH tolerance of 5–8, and both enzymes retain thermostability after prolonged incubation at 60 °C [10]. The flexible lid domain enhances substrate-binding pocket accessibility, allowing efficient engagement with both polymeric and oligomeric substrates [10]. End-product profiles confirm chain scission and progressive depolymerisation, yet activity declines sharply against high-crystallinity commercial polyethylene terephthalate grades, revealing a persistent substrate-specificity limitation [11]. These kinetic advantages position archaeal enzymes as promising candidates for high-temperature or anaerobic polyester processing, although systematic comparisons under combined hydrostatic pressure and salinity remain scarce [18]. Fig. 2 shows the kinetic advantages of archaeal PETases in polyextreme environments. Data derived from Perez-Garcia et al. [10] and Acosta et al. [11]; error bars represent standard deviation of triplicate assays.

### 5.3 Hypothetical and indirect mechanisms for polyolefin degradation

Direct evidence for archaeal enzymes mediating efficient carbon–carbon backbone cleavage in polyolefins remains absent [16]. Hypothetical oxidative pathways analogous to bacterial laccases or peroxidases have been proposed, potentially enhanced by the inherent extremotolerance of archaea under high-pressure or strictly anaerobic regimes [18]. Surface alterations, biofilm-mediated radical generation, and limited mineralisation evidence, typically below 5 percent in most systems, constitute the primary observations reported to date [16]. Polyolefin hydrophobicity and high crystallinity further restrict enzyme accessibility, rendering abiotic and biotic attack inefficient in aphotic, high-salinity, or high-pressure niches [16]. Metagenomic surveys hint at hydrocarbonoclastic gene clusters within halophilic and methanogenic archaea, yet these clusters have not been functionally linked to polyethylene or polypropylene breakdown [35]. Critical evaluation of association versus causation in polyolefin-plastisphere communities is therefore essential, as many archaea may function as secondary colonisers rather than primary degraders [13]. These gaps underscore the need for targeted biochemical assays and synthetic biology approaches to test oxidative mechanisms under authentic extreme conditions [18].

### 5.4 Syntrophic contributions in anaerobic digestion systems

Methanogenic archaea contribute indirectly to polyester breakdown through syntrophic cross-feeding in anaerobic digestion systems [25]. Genera such as *Methanothermobacter* and *Methanosarcina* dominate thermophilic consortia, utilising fermentation intermediates and generating methane while accelerating overall polymer mineralisation [25,36]. Biodegradation rates vary markedly by polymer chemistry and temperature regime: polylactic acid and polyhydroxybutyrate achieve 53–95% conversion under thermophilic conditions with high methane yields, whereas polybutylene adipate terephthalate and polyethylene show negligible mineralisation [25,37]. Community shifts and morphological polymer erosion are consistently observed in reactors containing archaea, confirming their role in the methanogenesis phase [25]. Table 3 summarises these data and emphasises the disparity between readily degradable aliphatic polyesters and recalcitrant polyolefins. Engineered archaeal–bacterial consortia, therefore offer a promising route to enhance carbon conversion efficiency, although scalability is constrained by slow hydrolysis of high-crystallinity substrates and the requirement for precise control of anaerobic conditions [25].

**Table 3:** Anaerobic degradation rates of polyesters and polyolefins in AD systems (with/without archaea involvement).

Polymer	Conditions (temperature, duration)	Biodegradation rate (%)	Methane yield (NmL CH_4_/g VS or equivalent)	Archaeal involvement/key taxa	Key notes/mechanism	References
**Polylactic acid (PLA)**	Mesophilic 35–38 °C, up to 500 days	0–29 (often <5–10 in pure form; up to 80 with pretreatment)	Low (e.g., <50–100 in untreated)	Limited; methanogens secondary in syntrophy	Poor under mesophilic; slow hydrolysis; thermophilic enhances to 70–90% with particle reduction	Jin et al. [25]; Farveen et al. [37]; Álvarez-Méndez et al. [38]
**Polylactic acid (PLA)**	Thermophilic 55–60 °C, 60–100 days	53–95 (up to 90 in powder form)	200–400+ (enhanced with co-digestion)	Methanothermobacter dominant in thermophilic AD	Rapid hydrolysis at elevated temperature; lactate-utilising bacteria + methanogens	Yagi et al. [36]; Jin et al. [25]; Farveen et al. [37]
**Polybutylene adipate terephthalate (PBAT)**	Mesophilic 35–38 °C, 30–45 days	2–5 (low in pure form; blends variable)	Low (<50–100)	Minimal direct; syntrophic methanogens are possible	Poor degradability; aromatic units hinder; blends with PLA/starch show limited improvement	Farveen et al. [37]; Álvarez-Méndez et al. [38]
**Polybutylene adipate terephthalate (PBAT)/PLA blends**	Thermophilic, 45 days	Variable 20–70 (enhanced with pretreatment)	Increased with enzyme embedding (up to 5-fold)	Methanogenic archaea in AD communities	Thermophilic favors depolymerisation; pretreatment (enzymatic/thermal) boosts rates	Farveen et al. [37]; Cheng et al. [39];
**Polycaprolactone (PCL)**	Mesophilic 35–38 °C, 277 days	3–22	Not quantified	*Methanobacterium* petrolearium-like (95% similarity)	Surface erosion: low rate in mesophilic AD	Yagi et al. [36]
**Polyhydroxybutyrate (PHB)**	Mesophilic 35–38 °C, 9–14 days	80–90	High (rapid conversion)	Limited archaea; bacteria dominant (Arcobacter, Clostridium)	Fast degradation; bulk erosion; high methane potential	Yagi et al. [36]; Jin et al. [25]
**Polyhydroxybutyrate (PHB)**	Thermophilic, short periods	>80	High	Methanogenic involvement in syntrophy	Rapid under both regimes; known degraders	Yagi et al. [36]; Jin et al. [25]
**Polyethylene (PE)/Polypropylene (PP)**	Mesophilic/thermophilic AD	<1–5 (negligible)	Negligible	No direct involvement reported	Recalcitrant C-C backbone; no significant anaerobic breakdown; accumulation risk	Mohanan et al. [16]; Quecholac-Piña et al. [40].
**General biodegradable polyesters (e.g., PBS, TPS)**	Mesophilic/thermophilic AD	57–85 (mesophilic for some); 53–96 (thermophilic)	Variable 200–600+	*Methanothermobacter, Methanosaeta* in thermophilic; syntrophic roles	Bulk/surface erosion; archaea key in the methanogenesis phase	Jin et al. [25]; Falzarano et al. [41]

## 6 Comparative analysis: Archaea versus Bacteria and fungi in plastic degradation

Bacterial degraders predominate in aerobic, mesophilic environments, with genera such as *Pseudomonas*, *Bacillus*, *Rhodococcus*, and *Ideonella sakaiensis* achieving efficient polyethylene terephthalate depolymerisation via tandem PETase–MHETase systems or cutinases [5,8]. On low-crystallinity polyethylene terephthalate films under mesophilic conditions (30 °C, pH 7), *I. sakaiensis* achieves approximately 58 % weight loss over 42 days, yet performance declines sharply to below 10 % on high-crystallinity commercial substrates or polyolefins even after several months [5,6]. Fungal contributions involve extracellular oxidases (laccases, manganese peroxidases) that yield 1–40 % molecular-weight reduction on polyethylene under optimised laboratory incubation, although rates remain modest, substrate-specific, and dependent on prior photo-oxidative priming [7,8].

Archaea exhibit pronounced niche-specific advantages under polyextreme regimes. PET46 and GuaPA demonstrate hydrolysis rates on semi-crystalline polyethylene terephthalate powder comparable to or exceeding wild-type bacterial IsPETase and LCC at 70 °C, while releasing substantially higher quantities of bis(2-hydroxyethyl) terephthalate and mono(2-hydroxyethyl) terephthalate oligomers [10,11]. GuaPA further extends activity to low-crystallinity polyethylene terephthalate films, liberating measurable terephthalic acid within 48 h under thermophilic conditions where mesophilic bacterial enzymes rapidly lose activity [11]. These thermostable, halotolerant, and anaerobically functional enzymes retain activity in high-salinity or high-pressure matrices where bacterial and fungal counterparts are inactivated. Although extremophilic bacteria (such as deep-sea *Marinobacter* species) and fungi such as marine yeasts colonise plastispheres and achieve limited surface erosion, their documented efficiencies on recalcitrant polyolefins remain below 5 % mineralisation even after prolonged incubation, and their activity is further suppressed by combined stressors that archaea accommodate through specialised lid-domain architecture and metal-cofactor stabilisation [6,7]. Quantitative comparisons of microbial phyla abundance across environments are summarised in Table 4, illustrating the consistent bacterial dominance in aerobic niches alongside the context-dependent superiority of archaea in anaerobic, hypersaline, or high-pressure zones.

**Table 4:** Plastisphere archaeal vs bacterial abundance across environments.

Environment and study site	Plastisphere substrate and incubation period	Archaeal relative abundance percentage	Bacterial relative abundance percentage	Fungal relative abundance percentage	Key notes and dominant taxa	References
**Deep-sea Southwest Atlantic hadal zone 3300 m**	Polypropylene, high-density polyethylene, high-density polyethylene with oxo-degradable additives; 719 days in situ	1 to 7% phylum level; *Crenarchaeota* and *Nanoarchaeota* up to 7.3% on high-density polyethylene with oxo-degradable additives	Greater than 90 percent dominant phyla, Proteobacteria and Firmicutes	Not reported	Archaea contribute to the core microbiome despite low abundance; *Thaumarchaeota, Euryarchaeota,* and *Bathyarchaeota* are present; bacteria dominate the overall community	Agostini et al. [13]
**Deep-sea Rockall Trough, North-East Atlantic, approximately 2000 m**	Polystyrene, polyurethane; in situ deployment	Low, less than 5 percent specific archaeal taxa enriched, yet a minor fraction	Dominant greater than 90 percent Proteobacteria and Bacteroidetes	Not reported	Archaea are a transient or enriched minority; generalist bacteria comprise 92 percent of the community	Kelly et al. [24]
**General deep-sea plastisphere, various hadal and trench sites review synthesis**	Mixed macroplastic and microplastic	Typically less than 10 percent, often 1 to 5 percent in metagenomes	85 to 95 percent of Proteobacteria and Bacteroidetes are dominant	Less than 5 percent is rarely quantified	Archaea are more prominent in anaerobic and hydrothermal niches; bacteria and fungi dominate aerobic surfaces	Woodall et al. [14]; Sun et al. [42]
**Hypersaline basins and soils contaminated with hypersaline**	Hydrocarbon and plastic analog substrates; consortia	Up to 20 to 40 percent in halophilic consortia at the domain level at extreme salinity	60 to 80 percent of halophilic bacteria are co-dominant	Low, less than 5 percent	Archaea Halobacterium and Haloarcula enriched above 200 g/L salinity; higher archaeal fraction than in marine settings	Oren [9]; Martínez-Espinosa [15]; Lee et al. [30]
**Marine and seawater plastisphere non-extreme baseline comparison**	Various microplastics: polyethylene, polypropylene, polystyrene	Less than 1 to 5 percent is rarely dominant	90 to 98 percent Alpha-Gamma-Proteobacteria and Bacteroidetes	Variable up to 10 to 20 percent in some studies	Archaea minor component; fungi are more variable yet secondary to bacteria	Sun et al. [42]
**Soil plastisphere Sub-Saharan microplastic-impacted**	Microplastics in soil	Depleted in some archaeal families, Nitrososphaeraceae reduced	Dominant Actinobacteria and Proteobacteria enriched	Not quantified	Plastic-dependent archaeal depletion; bacteria are more responsive to microplastic presence	Rohrbach et al. [33]

Controversies, including cultivation bias that underestimates archaeal roles and challenges in proving causation versus association in complex communities, are now addressed with explicit reference to polyextreme synergies [18]. Synergistic interactions, whereby archaea supply cofactors or intermediates to bacterial or fungal partners, further enhance overall rates in extremes.

## 7 Case studies from extreme environments

In-situ and metagenomic studies are beginning to illuminate archaeal contributions across polyextreme plastispheres. Long-term deep-sea deployments (719 days at 3300 m depth) in the Southwest Atlantic Ocean demonstrated that *Thaumarchaeota*, *Euryarchaeota*, and *Bathyarchaeota* integrate into core microbiomes on polypropylene and high-density polyethylene despite representing only 1–7 % relative abundance [13]. Comparable archaeal enrichment, typically as minor components within Proteobacteria-dominated communities, has been observed in the Rockall Trough on polystyrene and polyurethane [24] and in Antarctic deep waters [26]. Functional metagenomics of Guaymas Basin hydrothermal sediments has provided direct enzymatic evidence: the lid-containing feruloyl esterase PET46 from *Candidatus* Bathyarchaeota hydrolyses semi-crystalline polyethylene terephthalate powder [10], while the subsequently discovered *Bathyarchaeia*-derived PETase GuaPA depolymerises low-crystallinity polyethylene terephthalate films [11].

Hypersaline basins strongly select for halophilic archaea such as *Halobacterium*, *Haloarcula*, and *Haloferax*. Halophilic consortia from plastic-contaminated salterns and hypersaline soils degrade polycaprolactone, polystyrene, and polypropylene at 15 % NaCl, with archaea enhancing biofilm resilience and syntrophic hydrocarbon metabolism [15,28].

In terrestrial extraterrestrial analogs (Antarctic Dry Valleys and Atacama Desert), extremotolerant archaea dominate microbiomes under conditions of desiccation, high radiation, and oligotrophy, providing promising models for plastic waste bioremediation in future space missions [3,31].

Collectively, these case studies illustrate the presence and emerging functional potential of archaea in extreme plastispheres. Nevertheless, the number of detailed investigations remains relatively small, highlighting the critical need for intensified exploratory basic research to generate additional robust case examples across diverse polyextreme settings and polymer types.

## 8 Novelty and emerging frontiers: Archaea as a paradigm shift for bioremediation

Archaea represent an untapped reservoir of extremozymes for recalcitrant polyolefin and polyester bioremediation, distinguished by adaptations to polyextreme conditions where bacterial and fungal systems exhibit limited efficacy [3,43]. Landmark enzymes such as PET46 and GuaPA introduce novel structural features, including flexible lid domains comprising three α-helices and two anti-parallel β-strands that enhance substrate access and confer thermostability up to 70 °C with broad pH tolerance (5–8), enabling hydrolysis of semi-crystalline polyethylene terephthalate powder at rates comparable to wild-type bacterial IsPETase and LCC while outperforming them on bis(2-hydroxyethyl) terephthalate and mono(2-hydroxyethyl) terephthalate oligomers [10,11]. These characteristics position archaeal hydrolases as complementary or superior for high-temperature, high-salinity, or anaerobic applications in deep-sea or hypersaline cleanup, where mesophilic bacterial enzymes rapidly lose activity.

Synergistic archaea–bacteria consortia further amplify degradation through metabolic cross-feeding, in which archaea supply cofactors, intermediates, or reducing equivalents under oxygen limitation, thereby accelerating overall polymer depolymerisation and mineralisation [25,44]. Biotechnological translation encompasses rational enzyme engineering for improved catalytic efficiency and solvent tolerance, alongside the construction of synthetic consortia tailored for extreme-condition reactors; recent advances in CRISPR-Cas genome editing of thermophilic and halophilic archaea have already demonstrated precise insertion of PETase modules, promising rapid optimisation of these systems [45,46]. Climate-action benefits arise from accelerated carbon cycling in extreme depositional sinks, potentially mitigating plastic-derived greenhouse gas emissions while contributing to blue-carbon strategies in hadal and hypersaline environments.

In extraterrestrial contexts, archaeal extremotolerance provides robust models for bioremediation of space-mission waste, supporting closed-loop life-support systems on Mars or lunar bases where radiation, desiccation, and microgravity impose severe constraints [31,47]. Astrobiology crossover perspectives further view plastic degradation as a proxy for organic breakdown in extraterrestrial regolith. Omics-driven discovery combined with directed evolution and CRISPR editing promises rapid advancement, positioning archaea as a paradigm shift toward resilient, multi-extreme bioremediation strategies. A biotechnological potential matrix for archaeal extremozymes across key extreme and space-related applications is presented in Table 5, highlighting differential maturity levels according to Technology Readiness Level (TRL), a standardised nine-point scale originally developed by NASA and now widely adopted in biotechnology and environmental engineering to assess the progression of innovations from basic principles observed (TRL 1) to actual system proven in an operational environment (TRL 9).

**Table 5:** Biotechnological potential matrix for archaeal extremozymes in bioremediation applications.

Target environment/application	Key archaeal advantages	Demonstrated applications or close analogs	Major limitations/barriers	Technology readiness level (TRL)	Priority research needs	References
**Deep-sea/hadal trench bioremediation**	Thermostability up to 70–80 °C; pressure-adapted folding; anaerobic capability; lid-domain enhanced substrate access	PET46 and GuaPA hydrolyse PET powder/film at 70 °C; *Bathyarchaeota* in deep-sea plastispheres	No in-situ demonstration; low enzyme yield from uncultured sources; high-pressure reactor design complex	2–3 (conceptual proof; lab-scale enzyme activity)	In-situ mesocosm trials at hadal depths; high-pressure enzyme kinetics; metagenomic mining for pressure-adapted variants	Perez-Garcia et al. [10]; Acosta et al. [11]; Agostini et al. [13]
**Hypersaline basin/salt-lake cleanup**	Extreme halotolerance (>200–300 g/L NaCl); compatible solute production; robust extracellular polymeric substances	Halophilic consortia degrade hydrocarbons (82–93% reduction in 12 months); broad organic bioremediation potential	No direct PET/polyolefin degradation data; osmotic stress may inhibit non-halophilic co-degraders; enzyme secretion is limited	3–4 (proof-of-concept in analog hypersaline soils)	Characterisation of halophilic esterases/oxidases on plastics; halophilic synthetic consortia; salinity-gradient reactor tests	Martínez-Espinosa [15]; Lee et al. [30]; Rezaei et al. [34]; Oren [9]
**Extraterrestrial analog sites/space mission waste management**	Radiation tolerance; desiccation resistance; low-water activity activity; psychro- and thermotolerance	Extremotolerant archaea in Atacama/Antarctic dry valleys; models for closed-loop bioreactors on Mars/lunar bases	No direct plastic degradation studies in analogs; radiation effects on enzymes unknown; microgravity/closed-system constraints	1–3 (analog-site microbiome characterisation; conceptual space application)	Enzyme stability under simulated Martian radiation/UV; low-gravity bioreactor prototypes; astrobiology-plastic crossover experiments	Cowan et al. [3]; Coleine et al. [31]
**General extreme-condition consortia engineering**	Syntrophic interactions with bacteria; methanogenic support in anaerobic zones; broad metabolic versatility	Anaerobic digestion consortia with methanogens enhance polyester breakdown; potential for co-cultures in extremes	Slow rates; cultivation difficulties; regulatory hurdles for engineered archaea in open environments	2–4 (lab-scale syntrophy demonstrated in AD)	CRISPR-edited archaeal strains; omics-guided consortia design; pilot-scale extreme bioreactors	Jin et al. [25]; Pérez-García et al. [18]; Cowan et al. 3
**Climate-action integrated bioremediation**	Enhanced carbon cycling in extreme sinks; potential to reduce plastic-derived CH_4_/CO_2_ emissions	Indirect via methanogenic pathways in AD analogs; deep-sea/hypersaline carbon sequestration potential	Long timescales; uncertain global flux impact; no life-cycle assessment for archaeal systems	1–2 (conceptual linkage to climate mitigation)	Life-cycle and greenhouse-gas modelling of archaeal bioremediation; integration with blue carbon strategies	Jin et al. [25]; Cowan et al. 3; Mohanan et al. [16]

### 8.1 Biodesign theory and application: the Polyextreme Archaeal Synthrophy Framework

The synthesis presented in this review gives rise to a new biodesign theory termed the Polyextreme Archaeal Synthrophy Framework. This framework posits that the most effective and resilient plastic bioremediation systems are not single-enzyme or single-species solutions but modular archaeal–bacterial consortia in which archaea serve as the foundational catalytic and environmental-stabilisation chassis. Three integrated design principles underpin the framework. First, lid-domain promiscuity, exemplified by the flexible α/β-hydrolase lid of PET46 and GuaPA, enables substrate access under high pressure, salinity, or low water activity. Second, syntrophic cross-feeding allows archaea to supply cofactors, reducing equivalents, or fermentation intermediates to bacterial partners, thereby sustaining complete mineralisation. Third, polyextreme robustness incorporates thermostability, halotolerance, radiation resistance, and anaerobiosis as core chassis traits rather than secondary modifications.

**Fig. 3:**
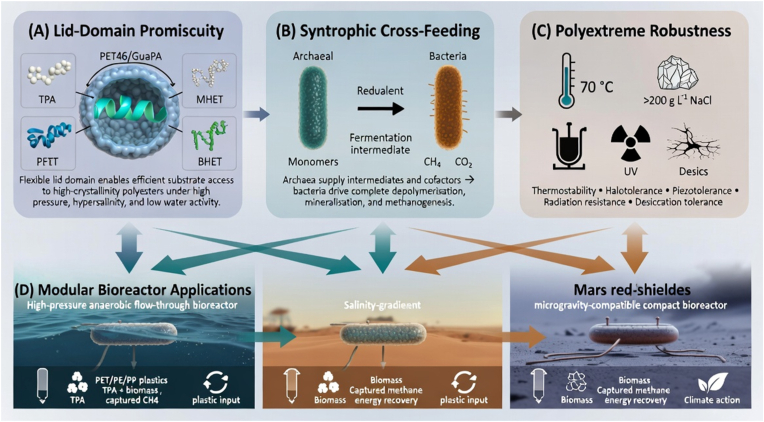
The Polyextreme Archaeal Syntrophy Framework: A Biodesign Strategy for Plastic Bioremediation in Extreme and Extraterrestrial Environments. The hierarchical infographic illustrates the integration of three core design principles: (A) lid-domain promiscuity of archaeal extremozymes (PET46 and GuaPA) enabling efficient polymer and oligomer access under polyextreme conditions, (B) syntrophic cross-feeding with bacterial partners (exchange of hydrolytic products, cofactors, and reducing equivalents for complete mineralisation and methanogenesis), and (C) inherent polyextreme robustness (thermostability up to 70 °C, halotolerance >200 g L^−1^ NaCl, anaerobiosis, pressure adaptation, radiation resistance, and desiccation tolerance). These principles underpin (D) modular bioreactor applications in hadal trenches (high-pressure anaerobic flow-through units), hypersaline basins (salinity-gradient bioreactors), and extraterrestrial/space habitats (radiation-shielded microgravity-compatible modules), yielding terephthalic acid, biomass, and energy recovery via captured CH_4_ while advancing circular-economy and climate-action objectives. (Created in BioRender. Chigwada, A. (2026) https://BioRender.com/3qy3358).

From this theoretical foundation emerges a practical bio-design application: the Extremophile Plastic Bioreactor Platform (Fig. 3). The platform is a scalable, modular system optimised for polyolefin and polyester degradation in both terrestrial extreme environments and closed-loop space habitats. The platform employs CRISPR-Cas-edited archaeal chassis expressing evolved variants of PET46 and GuaPA with enhanced lid-domain flexibility for higher-crystallinity substrates. These archaea are co-cultured with syntrophic bacterial partners to achieve synergistic depolymerisation and mineralisation. Reactor configurations are tailored to target environments: high-pressure anaerobic flow-through units for hadal deployment, salinity-gradient bioreactors for hypersaline basins, and compact, radiation-shielded microgravity-compatible modules for Mars or lunar life-support systems.

Projected performance, extrapolated from existing kinetic data, includes more than 70 percent PET powder and film degradation within 48 h at 70 °C and broad pH, with measurable surface erosion of polyolefins through archaeal oxidative gene clusters. Methane generated by methanogenic archaea can be captured for on-site energy recovery, thereby closing the carbon loop and contributing to climate-action objectives. Technology readiness is currently at TRL 2–3, and advancement to TRL 4–5 within two to three years is feasible through directed evolution of lid domains, synthetic consortium assembly, and in-situ mesocosm validation in hadal trenches, hypersaline salterns, and Antarctic dry-valley analogs.

By translating the review's enzymatic, community, and polyextreme insights into a coherent biodesign framework, the Polyextreme Archaeal Synthrophy Framework and the Extremophile Plastic Bioreactor Platform provide a blueprint for moving beyond incremental mesophilic solutions toward resilient, multi-extreme bioremediation strategies. This approach not only addresses current evidentiary gaps in polyolefin mineralisation but also aligns archaeal biotechnology with circular-economy principles and sustainable waste management both on Earth and in extraterrestrial settings.

## 9 Challenges, controversies, and future directions

Significant methodological limitations continue to impede definitive demonstration of archaeal causation in plastic degradation processes. A substantial proportion of current evidence derives from metagenomic analyses of plastisphere communities rather than from isolated strains or rigorously characterised pure enzyme kinetics [16]. This reliance on sequence-based inference frequently precludes direct attribution of observed polymer modifications to specific archaeal taxa or enzymes, while inconsistencies in sampling protocols, incubation durations, and analytical methods (for example, surface imaging versus quantitative mass-loss measurements) introduce further variability and reduce reproducibility across studies [42]. Cultivation of extremophilic archaea remains particularly challenging; many lineages exhibit strict requirements for high hydrostatic pressure, extreme salinity, or strict anaerobiosis that are difficult to replicate in standard laboratory settings, systematically underestimating archaeal diversity and functional potential within extreme plastispheres [7,48].

Research in this domain demands highly specialised scientific expertise and infrastructure. Investigators require proficiency in extreme-environment microbiology, high-pressure cultivation systems, metagenomic bioinformatics, enzyme crystallography, and synthetic biology. Access to specialised equipment for simulating hadal pressures or hypersaline conditions is not widespread, restricting the pace of experimental validation and necessitating large interdisciplinary teams that combine expertise from bioengineering, astrobiology, and materials chemistry [3,15].

Evidence gaps persist regarding complete mineralisation of polyolefins, with most reports confined to surface alterations, biofilm formation, or partial hydrolysis of polyesters. Association-versus-causation debates remain unresolved, as many archaea detected in plastispheres may function as secondary colonisers benefiting from primary degradation products rather than acting as primary degraders [13,49]. Slow degradation rates, low recombinant enzyme yields from uncultured lineages, and the inhibitory effect of high substrate crystallinity further constrain industrial scalability. Additionally, ethical and regulatory concerns surround the deployment of genetically engineered archaea in open marine or hypersaline environments and especially in closed-loop space habitats, where unintended ecological consequences or biosafety risks must be carefully evaluated [35].

Future research directions should prioritise high-throughput metagenomics coupled with single-cell omics and advanced multi-omics approaches (metatranscriptomics, metaproteomics) to bridge the gap between genetic potential and actual enzymatic activity. In particular, exploratory basic research must be intensified to generate additional case examples through systematic plastisphere surveys across under-sampled hadal, hypersaline, and analog sites, extended in-situ incubation experiments under authentic polyextreme conditions, and rigorous functional assays that establish causation rather than correlation. Rational protein design and directed evolution of archaeal hydrolases are essential to improve catalytic efficiency on high-crystallinity polyolefins and to enhance performance under polyextreme conditions. In-situ mesocosm experiments deployed in hadal trenches and hypersaline basins will be critical for validating community-level contributions under authentic environmental parameters. Interdisciplinary integration with policy makers will support the development of robust regulatory frameworks for safe deployment of archaeal biotechnologies in cleaner environmental systems, including extraterrestrial waste management. Ultimately, these advances could position archaea-driven bioremediation as a cornerstone of sustainable circular material economies in both terrestrial extremes and space exploration contexts.

## 10 Conclusion

Archaea-driven plastic degradation in extreme environments represents a transformative frontier in polyolefin and polyester bioremediation. This review has synthesised the distribution and persistence of recalcitrant polymers in hadal trenches, hypersaline basins, and extraterrestrial analog sites (Sections 3), characterised archaeal plastisphere communities (Section 4), detailed the metagenomic discovery and structural innovations of PET46 and GuaPA (Section 5), compared archaeal advantages with bacterial and fungal systems (Section 6), examined representative case studies (Section 7), and outlined novelty, challenges, and future directions (Sections 8, 9). The evidence demonstrates that archaea, long overlooked in plastic biodegradation research, possess unique extremozymes and metabolic versatility that confer robustness under polyextreme conditions where conventional microbial strategies falter.

The landmark enzymes PET46 and GuaPA illustrate this potential. Their flexible lid domains and Zn^2+^-dependent catalysis enable efficient hydrolysis of semi-crystalline PET powder and films at 70 °C and broad pH ranges, outperforming benchmark bacterial PETases on oligomers while retaining activity in high-salinity and anaerobic matrices. Syntrophic contributions of methanogenic archaea in anaerobic digestion further amplify polyester mineralisation, although direct polyolefin backbone cleavage remains elusive. These findings position archaea as complementary, and in many niches superior, partners in microbial consortia for deep-sea, hypersaline, and space-mission waste management.

From a climate-action perspective, accelerated archaeal mineralisation in extreme depositional sinks could reduce sequestered carbon pools and mitigate plastic-derived greenhouse-gas emissions. In extraterrestrial contexts, extremotolerant archaea offer practical models for closed-loop bioremediation on Mars or lunar bases, where radiation, desiccation, and microgravity impose severe constraints. The biotechnological potential matrix (Table 5) highlights technology readiness levels ranging from conceptual proof (TRL 1–3) to proof-of-concept consortia (TRL 3–4), underscoring the urgency of in-situ mesocosm trials, CRISPR-edited strains, and life-cycle assessments.

Persistent challenges, cultivation bias, metagenomic inference without proof of causation, slow rates on high-crystallinity substrates, and regulatory hurdles for engineered archaea must be addressed through high-throughput omics, rational enzyme design, and interdisciplinary policy frameworks. Filling these gaps will require sustained investment in extreme-environment microbiology and synthetic biology.

In summary, archaea-driven plastic degradation moves beyond incremental advances in mesophilic bacterial systems to deliver resilient, multi-extreme solutions aligned with circular-economy principles and global sustainability goals. By harnessing the untapped catalytic repertoire of polyextremophiles, this emerging paradigm can contribute meaningfully to cleaner environmental systems on Earth and in space. Future research must now translate laboratory discoveries into scalable, ethically governed applications, ensuring that the next decade witnesses archaea assuming their rightful place as cornerstone agents of plastic bioremediation.

## Declaration of generative AI and AI-assisted technologies in the writing process

Generative artificial intelligence (AI) tools were not used in the conceptualization, data analysis, interpretation, or writing of this manuscript. All content was produced solely by the named authors. The generative artificial intelligence (AI) tool (BioRender AI) was used solely for the design and high-resolution rendering of Fig. 1, Fig. 3. No AI tools were used for any other part of the manuscript.

## Author contributions

**Aubrey Dickson Chigwada:** Conceptualization, Methodology, Software, Validation, Formal analysis, Investigation, Resources, Data curation, Writing – original draft, Writing – review & editing, Visualization, Supervision. **Memory Tekere:** Conceptualization, Resources, Writing – review & editing, Visualization, Supervision, Project administration, Funding acquisition.

## Declaration of competing interest

The authors declare that they have no known competing financial interests or personal relationships that could have appeared to influence the work reported in this paper.

## References

[1] Ritchie H. (2023). Plastic pollution. Our World in Data.

[2] Peng G., Bellerby R., Zhang F. (2020). The ocean's ultimate trashcan: hadal trenches as major depositories for plastic pollution. Water Res..

[3] Cowan D.A., Ramond J.B., Maggs-Kölling G. (2024). Extremophiles in a changing world. Extremophiles.

[4] Zettler E.R., Mincer T.J., Amaral-Zettler L.A. (2013). Life in the “plastisphere”: microbial communities on plastic marine debris. Environ. Sci. Technol..

[5] Yoshida S., Hiraga K., Takehana T. (2016). A bacterium that degrades and assimilates poly(ethylene terephthalate). Science.

[6] Lv S., Li H., Wang Y. (2024). Biodegradation of typical plastics: from microbial diversity to metabolic mechanisms. Int. J. Mol. Sci..

[7] Atanasova N.S., Pietilä M.K., Oksanen H.M. (2021). Plastic degradation by extremophilic bacteria. Int. J. Mol. Sci..

[8] Danso D., Chow J., Streit W.R. (2019). Plastics: environmental and biotechnological perspectives on microbial degradation. Appl. Environ. Microbiol..

[9] Oren A. (2015). Halophilic microbial communities and their environments. Curr. Opin. Biotechnol..

[10] Perez-Garcia P., Chow J., Costanzi E. (2023). An archaeal lid-containing feruloyl esterase degrades polyethylene terephthalate. Commun. Chem..

[11] Acosta D.J., Barth D.R., Bondy J. (2025). Plastic degradation by enzymes from uncultured deep sea microorganisms. ISME J..

[12] Abel S.M., Wu F., Primpke S., Gerdts G., Brandt A. (2023). Journey to the deep: plastic pollution in the hadal of deep-sea trenches. Environ. Pollut..

[13] Agostini L., Moreira J.C.F., Bendia A.G., Kmit M.C.P., Waters (2021). Deep-sea plastisphere: long-term colonization by plastic-associated bacterial and archaeal communities in the Southwest Atlantic Ocean. Sci. Total Environ..

[14] Woodall L.C., Jungblut A.D., Hopkins K., Hall A., Robinson L.F., Gwinnett C. (2018). Deep-sea anthropogenic macrodebris harbours rich and diverse communities of bacteria and archaea. PLoS One.

[15] Martínez-Espinosa R.M. (2024 Jun 29). Halophilic archaea as tools for bioremediation technologies. Appl. Microbiol. Biotechnol..

[16] Mohanan N., Montazer Z., Sharma P.K., Levin D.B. (2020). Microbial and enzymatic degradation of synthetic plastics. Front. Microbiol..

[17] Chamley A., Baley C., Matabos M., Vannier P., Sarradin P.M., Freyermouth F., Davies P. (2024 Dec 20). Polymer material biodegradation in the deep sea. A review. Sci. Total Environ..

[18] Pérez-García P., Chow J., Costanzi E. (2025). It is dangerous to go alone: strategies to optimize PET depolymerization. Microb. Biotechnol..

[19] Olam M. (2023). Mechanical and thermal properties of HDPE/PET microplastics, applications, and impact on environment and life. Environ. Sci. J. Integr. Environ. Res..

[20] García-Martínez J.M., Collar E.P. (2022). The variance of the polypropylene α relaxation temperature in iPP/a-PP-pPBMA/Mica composites. J. Compos. Sci..

[21] Arhant M., Le Gac P.Y., Le Gall M. (2023). Non-Arrhenian hydrolysis of polyethylene terephthalate – a 5-year long aging Study above and below the glass transition temperature. Polym. Degrad. Stabil..

[22] Jian J., Xiangbin Z., Huang X. (2020). An overview on synthesis, properties and applications of poly(butylene-adipate-co-terephthalate)–PBAT. Adv. Ind. Eng. Polym. Res..

[23] Ardabili T.F., Kassaun B.B., Roohollahi H., Kazemian H. (2026). Poly (butylene adipate-co-terephthalate) (PBAT) and functional blends: applications, performance tuning, and emerging directions *Materials and Designs*. https://www.sciencedirect.com/science/article/pii/S0264127526000857.

[24] Kelly M.R., Whitworth P., Jamieson A., Burgess J.G. (2022). Bacterial colonisation of plastic in the Rockall Trough, North-East Atlantic: an improved understanding of the deep-sea plastisphere. Environ Pollut.

[25] Jin Y., Wang Y., Li H. (2022). Degradation of biodegradable plastics by anaerobic digestion: morphological, micro-structural changes and microbial community dynamics. Sci. Total Environ..

[26] Lacerda A.L., Kelly M.R., Dodhia M.S. (2025). Oceanic regions shape the composition of the Antarctic plastisphere. Commun. Earth Environ..

[27] Dodhia M.S., Kelly M.R., McGeehan J.E. (2023). Microbe-mineral interactions in the plastisphere: coastal and deep-sea insights. Front. Mar. Sci..

[28] Krumov N., Nikolova N., Ivanova V. (2025). New halophilic community degrades plastics. Fermentation.

[29] Varrella S., Li H., Wang Y. (2020). Deep hypersaline anoxic basins as untapped reservoir of polyextremophilic prokaryotes. Mar. Drugs.

[30] Lee C.E., Kelly M.R., Dodhia M.S. (2024). The primary molecular influences of marine plastisphere communities. Crit. Rev. Environ. Sci. Technol..

[31] Coleine C., Delgado-Baquerizo M., Rosado A.S. (2025). The role of extremophile microbiomes in terraforming Mars. Commun. Biol..

[32] Biagioli F., Bay S., Zerboni A., Coleine C. (2025). Caves on Earth as proxies for Martian subsurface environments. Int. J. Astrobiol..

[33] Rohrbach S., Li H., Wang Y. (2025). Microplastic impacts archaeal abundance, microbial networks and functional guilds in Sub-Saharan soils. FEMS (Fed. Eur. Microbiol. Soc.) Microbiol. Ecol..

[34] Rezaei F., Li H., Wang Y. (2025). Discovery and biodegradation characterization of halophilic plastic-degrading bacteria. Front. Microbiol..

[35] Krzmarzick M.J., Taylor D.K., Fu X. (2018). Diversity and niche of Archaea in bioremediation. Archaea.

[36] Yagi H., Ninomiya F., Funabashi M., Kunioka M. (2013). Thermophilic anaerobic biodegradation test and analysis of eubacteria involved in anaerobic biodegradation of four specified biodegradable polyesters. Polym. Degrad. Stabil..

[37] Farveen M.S., Muñoz R., Narayanan R. (2025). Enhancing bioplastic degradation in anaerobic digestion: a review of pretreatment and co-digestion strategies. Polymers.

[38] Álvarez-Méndez S.J., Ramos-Suárez J.L., Ritter A., Mata González J., Camacho Pérez Á. (2023). Anaerobic digestion of commercial PLA and PBAT biodegradable plastic bags: Potential biogas production and 1H NMR and ATR-FTIR assessed biodegradation. Heliyon.

[39] Cheng Y., Bi D., Zhang T., Kong Z., Jiang X., Wang S., Chen M., Shen Z., Zhang Y. (2023). Anaerobic co-digestion of PBAT/PLA/starch commercial bio-plastic bags with food waste. Biochem. Eng. J..

[40] Quecholac-Piña X., Hernández-Berriel M.D.C., Mañón-Salas M.D.C. (2020). Degradation of plastics under anaerobic conditions: a short review. Polymers.

[41] Falzarano F., Jin Y., Wang Y. (2023). Anaerobic biodegradability of commercial bioplastic products: systematic bibliographic analysis and critical assessment of the latest advances. Materials.

[42] Sun Y., Li H., Wang Y. (2023). Plastisphere microbiome: methodology, diversity, and functionality. iMeta.

[43] Sysoev M., Grötzinger S.W., Renn D., Eppinger J., Rueping M., Karan R. (2021). Bioprospecting of novel extremozymes from Prokaryotes—The advent of culture-independent methods. Front. Microbiol..

[44] Zhou Y., Zeeshan Ul Haq M. (2025). Engineering of synthetic microbial consortia for sustainable management of wastewater and polyethylene terephthalate: a comprehensive review. Int. J. Mol. Sci..

[45] Ye J.W., Li H., Wang Y. (2023). Synthetic biology of extremophiles: a new wave of biomanufacturing. Trends Biotechnol..

[46] Wang J., Wei J., Li H., Li Y. (2022). High-efficiency genome editing of an extreme thermophile Thermus thermophilus using endogenous type I and type III CRISPR-Cas systems. mLife.

[47] Koehle A.P., Brumwell S.L., Seto E.P., Lynch A.M., Urbaniak C. (2023). Microbial applications for sustainable space exploration beyond low Earth orbit. NPJ Microgravity.

[48] Rafiq M., Hassan N., Rehman M., Hayat M., Nadeem G., Hassan F., Iqbal N., Ali H., Zada S., Kang Y., Sajjad W., Jamal M. (2023). Challenges and approaches of culturing the unculturable archaea. Biology.

[49] Talukdar A., Banerjee A., Dey S., Bhattacharya S. (2024). The good, the bad and the ugly: critical insights on the applications of microbes in microplastic degradation. Cambridge Prisms: Plastics.

